# From perception to action: Waste management challenges in Kassena Nankana East Municipality

**DOI:** 10.1016/j.heliyon.2024.e32438

**Published:** 2024-06-05

**Authors:** Kwame Anokye, Sumaila Asaah Mohammed, Portia Agyemang, Ahunoabobirim Bosompem Agya, Ebenezer Ebo Yahans Amuah, Stephen Sodoke, Edmund Kude Diderutua

**Affiliations:** a Department of Environmental Science, C. K. Tedam University of Technology and Applied Science, Navrongo, Ghana; b Institute for Technology and Resources Management in the Tropics and Subtropics, Technische Hochschule Köln, Germany; c Department of Engineering, Nordhausen University of Applied Sciences, Nordhausen, Germany; d Department of Environmental Science, Kwame Nkrumah University of Science and Technology, Kumasi, Ghana; e Department of Geomatic Engineering, Kwame Nkrumah University of Science and Technology, Kumasi, Ghana; f University for Development Studies, Tamale, Ghana

**Keywords:** Sustainable waste management, Environmental awareness, Waste disposal methods, Policy effectiveness, Community engagement

## Abstract

This study examines the perceptions and behaviours related to waste management (WM) among residents of the Kassena Nankana East Municipality in Ghana. A mixed-method approach was used to garner data from 394 household respondents, and the data was analysed using SPSS software. The findings reveal a weak correlation between educational attainment and effective waste management practices and a mildly positive correlation between waste awareness and the effectiveness of policies and legislation. Notably, a significant proportion of respondents preferred open burning (42.1 %) and dumping (18.8 %) as disposal methods, indicating critical areas for intervention. The study introduces a novel comprehensive analysis by integrating attitude formation, collective action, and institutional and rational choice theories to understand WM behaviours. This theoretical integration significantly contributes to the field, providing a heterogeneous understanding of the factors influencing WM practices. Furthermore, the research identifies key gaps in WM infrastructure and public engagement, offering innovative recommendations to address these challenges. This study's significant outputs include identifying specific community behaviours towards waste disposal and evaluating the effectiveness of existing WM policies. These findings provide actionable insights for policymakers and stakeholders to develop targeted interventions that promote sustainable WM practices. The study's contributions and recommendations are crucial for advancing environmental sustainability efforts in similar contexts, aligning with the Sustainable Development Goals.

## 1 Introduction

Waste management (WM), one of the world's critical environmental challenges, is noted for its detrimental implications for public health, economic development, and ecological sustainability (Wan et al. [1]; Adami and Schiavon [2]). Studies (Khan et al. [3]; Anokye et al. [4]; Agya et al. [5]) have associated the global acute WM challenges, particularly in the global south countries, with the rapid urbanisation, inadequate WM infrastructure and limited financial and human resources. As the human population continue to expand, so does waste generation. Global waste generation is predicted to be at a pace of 480 million metric tons annually by 2030 (Saqib [6]). These predictions place financial, infrastructural and skilled human resources constrain on most growing metropolitan regions, leading to ineffectiveness and inefficiencies in managing waste (Zhang et al. [7]). Ghana and Nigeria are among Africa's top waste-generating countries (Orhorhoro and Oghoghorie [8]). However, due to a lack of infrastructural and technical know-how, these countries suffer from ensuring effective and sustainable WM practices (Godfrey et al. [9]; Chisholm et al. [10]). In Ghana, in particular, communities continue to experience disproportionate waste collection, overflow from central waste bins, a lack of storage containers, and designated waste dumping sites (Asante et al., [11]. The disproportionate share of the municipal for WM technologies (towards sustainable waste collections, transportation, separation, recycling, resource recovery, and proper disposal) has encouraged indiscriminate household waste disposal, leading to most environmental and health concerns. Research (Wan et al. [1]; Wilson [12]) and WM policy frameworks (Knickmeyer [13]; Tobin and Zaman [14]) have advocated for all-inclusive WM programs to target and sustainably manage waste at the household levels. Raghu and Rodrigues [15] argue that household attitudes, perceptions, and behaviour can still shape the adoption of new and existing management technologies and policies. Nonetheless, behavioural patterns can be altered by education, raising awareness of the negative effects of specific behaviours, law enforcement and institution of regulatory measures that make some behaviours illegal (Minelgaitė and Liobikienė [16]). Although extensive studies have established the detrimental effects of improper WM in societies due to the absence of some basic facilities, including waste bins and designated dump sites, there is a scientific knowledge gap on how household behaviour and perception shape the adoption of actionable WM technologies and practices towards mitigating WM challenges. The Kassena Nankana East Municipality (KNEM) in the Upper East Region of Ghana exemplifies WM challenges and struggles with effectively managing solid waste, which endangers both the well-being of its residents and the environment. Although the KNEM is predominantly rural with an agricultural economy, the municipality has experienced significant population growth in recent years, leading to increased waste generation (Adabugah [17]; Pervarah et al. [18]). Aside from the shortfalls in its WM facilities, the demographic transformation of the KNEM has placed immense insistence on the existing WM systems, which are insufficient to handedly suppress the growing volumes of waste (GSS [19]). The resultant improper disposal and management of waste lead to environmental pollution, health issues, and a decline in the quality of life. One of the primary obstacles in addressing WM issues in KNEM is the gap between public perception and practical action. Household understanding of WM practices, their attitudes towards waste disposal, and their willingness to engage in sustainable behaviours significantly influence the effectiveness of WM strategies (Raghu and Rodrigues [15]). Knickmeyer [13] and Masoabi [20] also reported that misconceptions about waste segregation, lack of awareness about the environmental impacts of improper waste disposal, and limited community engagement are pervasive challenges that hinder the implementation of effective WM solutions. Therefore, addressing the varied WM challenges in KNEM needs the examination of the perceptions, adopted WM methods, and behaviours of its residents, the adequacy of existing municipal WM infrastructure, and the role of local governance (Venkataramanan et al. [21]) for a better and broader understanding of the subject matter. The novelty of this study lies in its comprehensive examination of waste management dynamics, integrating multiple theoretical frameworks to understand the reciprocal reaction and action between individual attitudes, rational, collective action, and institutional contexts. Only when the interplay between these factors is understood can we identify the critical areas for intervention and develop comprehensive strategies to bridge the gap between perception and action. More so, effective WM technologies and strategies coupled with planned behaviour and determined willingness can significantly extenuate Greenhouse Gas (GHG) emissions and contribute to climate change mitigation efforts (Wang et al. [1]; Shah et al. [22]). To achieve this objective, the study employed a mixed-methods approach (Timans et al. [23]), integrating quantitative surveys to quest household perceptions and qualitative interviews to obtain deeper insights into the attitudes and behaviours of different households and waste management companies (Stockemer et al. [24]). This holistic approach provided a robust basis for providing practicable and actionable recommendations to address the unique WM challenges faced by the KNEM and finally contribute to the Sustainable Development Goals (SDGs), particularly Goals 11 (Sustainable Cities and Communities), 12 (Responsible Consumption and Production) and 13 (Climate Action to reduce Climate Change).

## 2 Research methodology

### 2.1 Study area description

**Fig. 1:**
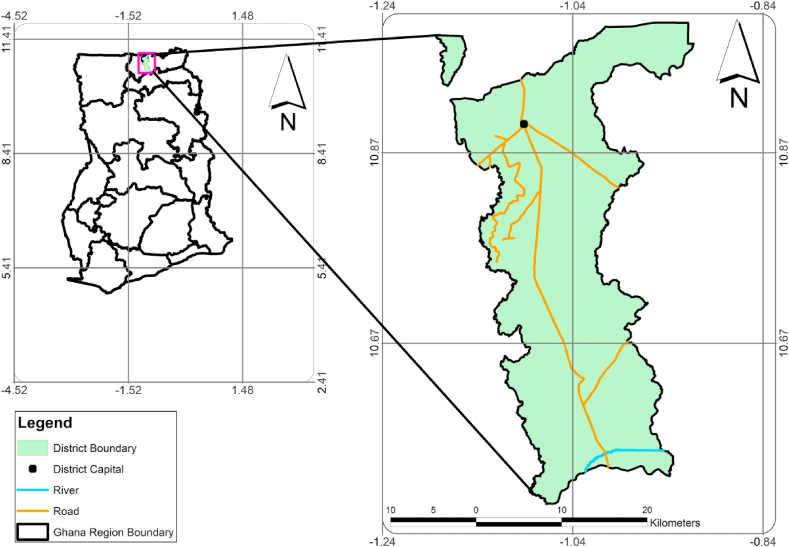
The map of Ghana showing Kassena Nankana East Municipal [Map designed with ArcMap 10.7].

The Kassena Nankana District, created in 1988 by LI 1855, was raised to the Kassena Nankana Municipal by LI 2106 (GSS, [25]; Anokye et al. [4]). The municipality is one of the thirteen municipalities in the Republic of Ghana's Upper East Region (Fig. 1). Navrongo is the municipality's seat of government and administration. Bordered by Navrongo to the north is Burkina Faso and the Kassena-Nankana-West District (Fig. 1). The Municipality faces significant WM challenges, including inadequate infrastructure and traditional disposal practices (Ampofo et al. [26]; Anokye et al. [4]). The population distribution in Navrongo is predominantly youth below 19 years old (GSS [25], GSS [27]). Islam et al. [28] stated that a population with youth constituting the majority could lead to higher consumption and waste generation rates, necessitating effective WM strategies. Therefore, from an effective policy institution standpoint, it was crucial to assess the WM facilities, adopted household WM technologies and recycling infrastructure that could hamper effective WM. Additionally, as the situation in the municipality reflected broader WM issues faced by many developing regions, highlighting the importance of theory-based comprehensive strategies, infrastructure development, and community engagement in addressing these challenges is important to replicate in similar contexts.

### 2.2 Research approach

The study employed a mixed case study research design to examine the understanding of residents' perceptions and knowledge of waste in the environment. This research approach was used to enable the researchers to garner in-depth knowledge about the residents' preferred waste disposal methods, their satisfaction with the municipal waste disposal methods, the municipal's satisfaction and the companies' waste management policies. Additionally, this case study ensured that data was gathered from a various of sources encompassing quantitative (questionnaires) and qualitative (in-depth interviews with key informants and observations) elements. This harnessed the credibility and robustness of collecting both qualitative and quantitative data. The quantitative data included the sociodemographic characteristics, waste disposal methods, level of satisfaction with the municipal's waste disposal methods, and awareness of the environmental impact of trash on the surveyed households. Qualitative data were obtained through in-depth interviews regarding municipal authorities' and waste companies' perspectives on waste management practices, satisfaction levels, and their comprehension of waste's environmental impact.

### 2.3 Study population

The Municipality has six (6) Zonal councils: Navrongo Zonal Council, Doba Zonal Council, Manyoro Zonal Council, Pungu Zonal Council, Kologo Zonal Council and Naaga Zonal Council (Fig. 1). The Zonal Councils serve as hubs for community enthusiasm surrounding the Local Government system. Council members are in charge of running these local governments. Additionally, it is separated into 34 Unit Committees and 35 Electoral Areas. Unit Committee: Kugongo/Kulongo Electoral Area does not have one. These Unit Committees are important in enforcement and mobilisation matters since they are closer to the people. Since the study will concentrate on the Kassena Nankana East Municipality, the target population is all the households in the Municipality.

### 2.4 Source of data, sampling technique and sample size determination

The study's data came from primary as well as secondary sources. Primary data was effectively collected by giving surveys to participants in their homes, important informants in the municipality, and private waste management firms. Conversely, secondary data is obtained through journals, publications, and any additional pertinent government records from the municipality's waste management unit. Several data sources were used to guarantee the accuracy of the information gathered (Sodoke et al. [29]).

The participants in this research were chosen using several sampling strategies, which included systematic, purposeful, and accidental samples. The Municipal was zoned or stratified into six zones as restricted by the Municipal. Purposive sampling was used to select all the communities in the six zonal councils since the researcher wanted to consider each individual in the community. This was purposively done as they represent the entire municipality. Dumping solid waste in open spaces is a common practice in urban areas, where 43.6 practice this method of waste disposal (GSS [25]). Again, as the Municipal Waste Officers indicate, the selected selected electoral areas were confirmed as having indicators of waste challenges. From the purposive sampling, a systematic sampling technique was used to select houses in each stratum (selected electoral areas in the zones). The researcher divided the average total number of households (24,365) of the zonal councils of the municipality by the sample size (394) (Essaw and Sasu [30]). This yielded a figure of 62, representing the interval from which all 394 households were sampled and the respondents were taken.

Respondents were selected randomly. The population size included all the selected households in the zonal councils of the Kassena Nankana East Municipality. The total number of households in these sampled areas is 24,365 (and the average household in the municipality is 4.1). Therefore, the sample size (394) for this study is calculated using the formula described by Puopiel [31]:

(1) $$n=\frac{N}{1+N{\left(e\right)}^{2}}$$

Where n = the sample size, N = the total households in the zonal councils and e = margin of error (0.05) at a confidence interval of 95 %. The statistical method employed for choosing the sample size guaranteed the reduction of mistakes in the data-gathering process (Puopiel [31]). Every community within the zonal council received a portion of the expected sample size. Because they represented the municipality's primary subdivisions, each community served by the zonal councils was chosen on purpose (Ampofo et al. [26]). Following the population estimations of the communities during the study year, the sample size of 394 households were further divided into a ratio representation, yielding a factor of 0.01617(394/24365 = 0.01617) per household in each community. The socioeconomic survey results were analysed according to the population sample to provide an equitable portrayal of the municipality.

Depending on how many sample units there are in each community (Table 1), fifteen (15) households were selected from Pungu Zonal Council, from the Doba Zonal Council, thirty-eight (38) households, from the Navrongo Zonal Council two-hundred and fifty households (250), twenty-seven (27) households from the Kologo Zonal Council, fifteen (15) households from Naaga Zonal Council and forty-eight (48) households from Manyoro Zonal Council. The total population, average households and sample size of the zonal councils are summarised in Table 1.

**Table 1:** Population and sample size of electoral area in the zonal councils.

Zonal Council	Population	Households	Sampling	Sample size
Pungu Zonal Council	3805	928	928 x (0.01617)	15
Doba Zonal Council	9733	2374	2374 x (0.01617)	38
Navrongo Central Council	63478	15482	15482 x (0.01617)	250
Kologo Zonal Council	6876	1677	1677 x (0.01617)	27
Naaga Zonal Council	3769	919	919 x (0.01617)	15
Manyoro Zonal Council	12,234	2984	2984 x (0.01617)	48
	99895	24,365	24365 x (0.01617)	394

### 2.5 Data collection instruments

Participants in the study were given questionnaires to complete to collect primary data. The questionnaire was divided into five pieces. Section A asked questions about the participant's gender, year of birth, work, schooling, and duration of residence in the municipality. Section B solicited information on household waste disposal methods and preferred waste methods. Section C discusses households' satisfaction with waste disposal methods by the municipality. Section D talks about households' knowledge/awareness of the impact of waste on the environment. Lastly, section E solicits information from key informants. The questionnaire was either provided via a Google link or manually delivered, then collected from the participants' homes. There was ample time for those who participated to complete the surveys. According to the researchers, this approach will allow the participants adequate time for introspection, focus, and, in some instances, consultation. To ensure adequate comprehension and responses from the semiliterate participants, the questions were made available in their native tongues. Personal interviews were carried out with important informants such as the Municipal Environmental Health Officer and the Zoomlion Municipal Supervisor. The investigator revealed the aim of the investigation. Participants were informed that the purpose of the interviews was not to assess their expertise but rather to gather information for the researcher. Data was also gathered through inspection. The researchers documented examples of subpar waste management in the town through observation. Using a camera, the researchers took images of the waste strewn all over the municipality and dumping sites.

### 2.6 Data processing and analysis

The SPSS software version 20 generated codes after the input of the primary data. The findings were displayed using graphical representations and tables displaying frequency distributions and descriptive statistics. Utilising the Pearson Correlation Coefficient Test at the 0.01 and 0.05 significance level, the associations between solid waste management techniques and the environment and other demographic information and environmental perception were determined.

The range of the correlation coefficient, r, is −1.00 to 1.00. A positive linear association, where one variable increases as another does, is indicated by a value of r > 0. A negative longitudinal association, where one variable increases while the other declines, is shown by a value of r < 0. There cannot be a linear relationship between the variables if r = 0. As a result, the correlation coefficient's absolute value indicates how strongly the variables are related as they approach one and how weakly it does as they approach zero (Mundo et al. [32]). The severity index (SI) was derived using the formula found in Al-Hammed and Assaff [33] and expressed as responses on a 0-to-4-point Likert Scale.

(2) $$SI=\frac{\sum_{i-0}^{4}a_{1x_{1}}}{4\sum_{i-0}^{4}x_{1}}$$

Where $a_{1}$ = the class index, a quantity that represents the weight assigned to the class $x_{1}$ = frequency of teachers' responses

i = 0, 1,2,3,4 and outlined as follows: where:

x_0_, x_1_, x_2_, x_3_, x_4_ are the responses' frequencies in line with

a_o_ = 0, a_1_ = 1, a_2_ = 2, a_3_, = 3, a_4_ = 4, correspondingly.

The rating system was modified in response to Majid and McCaffer [34]:

a_0_ = Strongly disagree 0.00 ≤ SI < 12.5

a_1_ = Disagree 12.5 ≤ SI < 37.5

a_2_ = Neutral 37.5 ≤ SI < 62.5

a_3_ = Agree 62.5 ≤ SI < 87.5

a_4_ = strongly agree 87.5 ≤ SI < 100.

To determine the main themes determined by the individual interviews, the key informants, experts, and stakeholders who were interviewed using open-ended, unstructured questionnaires for the qualitative analysis were transcribed, read multiple times, and then arranged, analysed, and examined for connections, patterns, and sequences (Agya et al. [5]). After that, the results were laid out interpretively, with significant results, insights, and empirical support for debates provided via snippets from particular interviews. Each interview was compared with several interviews to prepare the data to identify trends and essential details that would help comprehend the dynamics of work-related stress in the areas under study.

## 3 Results and discussions

### 3.1 Social demographic characteristics

The socio-demographic backgrounds of respondents in the Kassena Nankana East Municipal are presented in Table 2. The results show that the majority of the respondents, which represent 54.6 %, were males as compared to 45.4 % were females. Gender differences may affect how individuals see waste management, according to Mintz et al. [35]. Gender differences can influence waste management in various ways, and it is important to recognise that. According to Padilla and Trujillo [36], cultural and social factors may influence the type of gender responsible for the types of waste generated, disposal methods and attitudes toward waste management. More females are responsible for managing waste in the municipality (Table 2), which is in line with studies conducted by Smith and Johnson [37] and Garcia et al. [38]. Women often play a significant role in household waste management and recycling activities (Fan et al. [39]). For instance, women might be more involved in household waste segregation, while men might be more engaged in industrial waste management (Knickmeyer [13]). Again, one gender might feel more responsible for waste management due to societal expectations. Gender-sensitive waste management approaches can help address waste management and promote more inclusive and sustainable practices (Sharma et al. [40]). Women commonly take charge of domestic waste management activities, including segregation, recycling, and composting (Nepal et al. [41]). Their involvement in waste management influences the household's overall waste reduction and recycling efforts. Most respondents (33.8 %) were between 31 and 40 years of age. Age is anticipated to have a substantial influence because maturation may impact one's degree of awareness regarding environmental health and sanitation (Fatimah et al. [42]). However, there is a weak inverse correlation between age and awareness (Table 3, *r* = *-*0*.146*). Younger generations might have different consumption patterns compared to older generations. With the rise of fast fashion, electronic gadgets, and disposable products, younger people might generate more waste due to higher consumption rates.

**Table 2:** Demographic characteristics.

Description		Frequency	Percentage
Gender of Respondents	Male	215	54.6 %
	Female	179	45.4 %
Age of Respondents	Below 20 yrs.	40	10.2 %
	20–30	120	30.5 %
	31–40	133	33.8 %
	41–50	71	18.0 %
	Above 50 yrs.	30	7.6 %
Level of Education	None	30	7.6 %
	Informal	29	7.4 %
	Basic	27	6.9 %
	Senior High School	66	16.8 %
	Tertiary	242	61.4 %
Marital Status	Single	202	51.3 %
	Married	192	48.7 %
Occupations of Respondents	Unemployed	42	10.7 %
	Farmer	52	13.2 %
	Informal/non-formal	13	3.3 %
	Business	73	18.5 %
	Student	60	15.2 %
	Professional	154	39.1 %
The number of years respondents have stayed in the Municipality.	Below 1 yr.	16	4.1 %
	1–5	130	33.0 %
	Above 5 yrs.	248	62.9 %
Identify your locality	Doba Zonal Council	42	10.7 %
	Kologo Zonal Council	26	6.6 %
	Manyoro Zona Council	46	11.7 %
	Naaga Zonal Council	15	3.8 %
	Navrongo Central Council	250	63.5 %
	Pungu Zonal Council	15	3.8 %
Waste management is a problem in this area.	No	73	18.5 %
	Yes	321	81.5 %
I am aware of the environmental health impact of poorly managed waste.	No	19	4.8 %
	Yes	375	95.2 %
Waste hurts environmental health	No	33	8.4 %
	Yes	361	91.6 %
Number of people in the household	0–1	15	3.8 %
	2–5	235	59.6 %
	Above 5	144	36.5 %
Who is responsible for the management of household waste?	Children	101	25.6 %
	Community	33	8.4 %
	Father	56	14.2 %
	Head of household	39	9.9 %
	Mother	165	41.9 %

**Table 3:** Pearson Correlations for some Different Parameters.

		Age	Awareness
Age	Pearson Correlation	1	−0.146^a^
	Sig. (2-tailed)		0.004
Awareness	Pearson Correlation	−0.146^a^	1
	Sig. (2-tailed)	0.004	
		Years they stayed in Municipality.	Awareness
Years they stayed in Municipality.	Pearson Correlation	1	0.025
	Sig. (2-tailed)		0.624
Awareness	Pearson Correlation	0.025	1
	Sig. (2-tailed)	0.624	
		Occupations	Willingness
Occupations	Pearson Correlation	1	0.248^a^
	Sig. (2-tailed)		0.000
Willingness	Pearson Correlation	0.248^a^	1
	Sig. (2-tailed)	0.000	
		Level of Education	Willingness
Level of Education	Pearson Correlation	1	0.380^a^
	Sig. (2-tailed)		0.000
Willingness	Pearson Correlation	0.380^a^	1
	Sig. (2-tailed)	0.000	
	N	394	394

a Correlation is significant at the 0.01 level (2-tailed).

Most of the respondents were educated, and a percentage of 61.4 % had tertiary education, against 7.6 % with no form of formal education. Education can help people grasp the broader environmental impact of their actions. Those with higher education levels are more likely to understand the long-term consequences of waste accumulation, pollution, and the depletion of natural resources. Education can influence behaviour change. Educated individuals are more likely to adopt sustainable practices, including waste reduction, recycling, and composting, due to their understanding of the benefits and willingness to contribute positively to society. From Table 3, even though there is a weak correlation between respondents' education level and willingness to adopt good waste management, the results still indicate a significant relationship between respondents’ education level and willingness to adopt good waste management practices.

Concerning the occupations of respondents, 39.1 % were professionals. Occupation can substantially influence waste management practices and behaviours (Govindan et al. [43]). Different occupations can shape an individual's waste generation patterns, awareness of environmental issues, and ability to adopt sustainable waste management practices (Table 3, *r* = *0.248*). Professionals working in office settings often generate waste from paper, packaging, and electronic equipment. Encouraging practices such as double-sided printing, digital document sharing, and proper e-waste disposal can help reduce waste in these environments. Most respondents, 62.9 %, have lived in the municipality for over five years. This indicates that the respondents gave an accurate picture of waste management strategies in their locality. Most respondents (81.5 %) agreed that waste management is a local problem. A more significant number (95.2 %) of the respondents concurred that they know the environmental health impact of poorly managed waste in their locality. In the Municipality, 41.9 % of persons responsible for managing household wastes were females (mothers), which is in line with a study conducted by Viljoen et al. [44].

The results presented in Table 3 show correlations between different variables (parameters). The Pearson correlation coefficient between the age of respondents and their awareness of the environmental health impact of poorly managed waste is −0.146, indicating a weak inverse relationship between age and awareness. Specifically, as the age of respondents increases, their awareness of the environmental health impacts of poorly managed waste tends to decrease slightly. The correlation is statistically significant with a p-value of 0.004, suggesting that the observed relationship is not due to random chance.

A study by Confetto et al. [45] and Dragolea et al. [46] found that younger generations, particularly millennials and Generation Z, are more likely to be engaged in environmental issues and advocate for sustainable practices. This trend is attributed to greater exposure to environmental education and activism through modern curricula and social media platforms. Similarly, Gray et al. [47] reported that environmental knowledge and concern are significantly higher among younger individuals, who are more likely to participate in environmental actions and support green policies. In contrast, older adults may have had less exposure to contemporary environmental education and may prioritise different issues based on their life experiences. This disparity in awareness levels across age groups highlights the need for targeted educational campaigns to raise awareness about the environmental health impacts of poorly managed waste among older populations. For example, community-based programs that incorporate environmental education tailored to older adults' preferences and communication styles could be effective. These programs could utilise local media, community centres, and intergenerational initiatives to disseminate information and encourage proactive waste management behaviours.

The Pearson correlation coefficient between the years respondents have stayed in the municipality and their awareness of the environmental health impact of poorly managed waste for this relationship is 0.025, indicating a very weak positive correlation. This implies that there is almost no relationship between the duration of residence in the municipality and the respondents' awareness of the environmental health impacts of poorly managed waste. Furthermore, the significance value (p-value) of 0.624 suggests that this correlation is not statistically significant, meaning that the weak correlation is likely due to random chance rather than a meaningful relationship. The weak and statistically insignificant correlation suggests that the length of time individuals have lived in the municipality does not significantly influence their awareness of the environmental health impacts of waste mismanagement. This finding indicates that simply living longer in the area does not inherently improve residents' understanding or awareness of environmental health issues.

Comparing these findings with recent research, it appears consistent with some studies but contrasts with others. For example, a study by Bennett et al. [48] found that long-term residency in a particular area did not significantly correlate with higher environmental awareness unless accompanied by active participation in community-based environmental programs. This suggests that merely residing in a place for an extended period does not guarantee increased environmental knowledge; instead, proactive engagement and education are necessary. Conversely, research by da Silva Costa et al. [49] highlighted that in specific communities, more extended residency was associated with higher environmental awareness due to accumulated local knowledge and experience with environmental issues. Over time, individuals become more familiar with local waste management infrastructure, regulations, and community norms. This discrepancy suggests that the relationship between residency duration and environmental awareness might vary depending on specific community dynamics and the presence of environmental education initiatives.

The Pearson correlation coefficient between respondents’ occupations and their willingness to adapt and adopt a favourable waste management system that will not adversely affect the environment and mitigate climate change is 0.248, indicating a weak to moderate positive relationship. This suggests that respondents' occupations are somewhat associated with their willingness to embrace environmentally friendly waste management practices. The correlation is statistically significant with a p-value of 0.000, meaning the relationship is highly unlikely to be due to random chance. These findings have important implications for the study, highlighting that occupational status shapes attitudes towards waste management and climate change mitigation. Individuals in certain occupations may have greater exposure to environmental education or workplace practices that promote sustainability, influencing their willingness to adopt favourable waste management systems. For instance, professionals or those engaged in environmentally focused industries may be more aware of the benefits of sustainable practices and more inclined to support and implement them.

The results are consistent with studies that show occupational influence on environmental attitudes and behaviours. For example, a study by Molnár et al. [50] found that employees in green industries or organisations with strong corporate social responsibility (CSR) policies are more likely to engage in pro-environmental behaviours at work and in their personal lives. This highlights how professional environments can significantly shape individuals' sustainability and waste management attitudes. Similarly, research by Schmitt et al. [51] indicated that occupational exposure to environmental issues can enhance environmental literacy and motivate individuals to adopt sustainable practices. Their study revealed that workers in sectors such as environmental science, education, and health services often exhibit higher levels of environmental concern and are more proactive in adopting green practices than those in other industries.

The Pearson correlation coefficient between the level of education and the willingness to adopt good waste management practices among the 394 participants is indicated by the value of 0.380, which signifies the strength and direction of the relationship between the two variables. A (weak) positive correlation coefficient (in this case, 0.380) suggests that as the level of education increases, the willingness to adopt good waste management practices also tends to increase. The significance level, represented by "Sig. (2-tailed)" with a value of 0.000, indicates the probability of obtaining the observed correlation coefficient by chance. A significance level of 0.000 indicates that the correlation is statistically significant at p < 0.001, meaning it's doubtful to have occurred by random chance alone. The significance of education as a tool for encouraging sustainable behaviours and solving environmental concerns is highlighted by the implications of the association between education level and willingness to embrace effective waste management techniques. By funding education and awareness campaigns, society can transition to more environmentally friendly waste management techniques and build a healthier environment for present and future generations.

The findings (Table 3) imply that raising people's knowledge and understanding of waste management techniques can favourably impact their behaviour. Programs for education aimed at various educational levels might contribute to raising awareness and comprehension of the significance of appropriate waste management. This finding is in line with that of Cavaliere et al. [52], who asserted that a person's beliefs or educational attainment might positively or negatively impact how they perceived management based on their degree of knowledge. Higher levels of education often lead to greater awareness of environmental issues, including the importance of proper waste management. Educated individuals are more likely to understand the consequences of improper waste disposal on the environment and human health (Minelgaitė and Liobikienė [16])

**Fig. 2:**
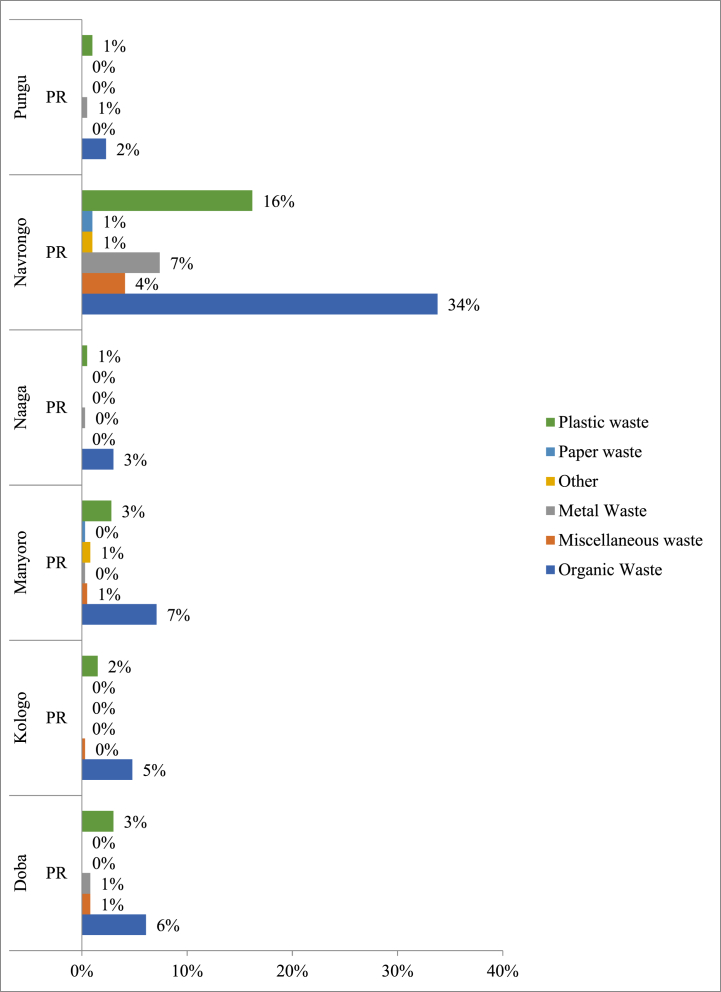
Dominant Waste Produced per Zonal Council, PR = Percentage responses.

The predominantly generated waste in each zonal area is presented in Fig. 2. From the results, it can be ascertained that most of the dominant wastes generated in each municipality are organic waste, followed by plastic waste and miscellaneous waste. At the same time, the least are metal waste and paper waste. The findings suggest several implications for promoting behavioural change towards sustainable waste management practices. Since organic waste is identified as the predominant waste type in each zonal area, there should be a strong emphasis on implementing composting programs and other organic waste management initiatives. Educating residents on the benefits of composting, providing resources for home composting, and establishing community composting facilities can help divert organic waste from landfills, reduce methane emissions, and create valuable compost for soil enrichment. This finding aligns with research by Kaur [53], who indicated organic waste makes up a sizeable portion of municipal solid trash. Kaur's study emphasises the significance of composting systems in keeping organic waste out of landfills and lowering greenhouse gas emissions. With plastic waste identified as the second most dominant waste type, efforts should be directed towards reducing single-use plastics, promoting recycling and upcycling of plastic materials, and implementing policies to encourage sustainable alternatives to plastics. Public awareness campaigns, plastic waste collection drives, and collaborations with businesses to reduce plastic packaging can all contribute to minimising plastic waste generation. However, according to a Maqsood et al. [54], plastic waste frequently overtakes organic waste as the most common type in high-density metropolitan regions. Variations in trash generation patterns and population density could cause this difference.

Miscellaneous waste diverse nature makes it challenging to manage effectively. To address this, there is a need for comprehensive waste sorting and recycling programs that target specific categories of miscellaneous waste, such as electronics, textiles, and hazardous materials. Providing convenient recycling drop-off points, organising community clean-up events, and raising awareness about disposal methods for miscellaneous waste can help reduce its environmental impact. Although metal and paper waste are identified as the least dominant waste types, they still contribute to environmental degradation when improperly disposed of. Encouraging residents and businesses to separate metal and paper waste for recycling, expanding recycling infrastructure, and offering incentives for recycling these materials can help increase recycling rates and conserve valuable resources. Studies highlight the difficulties in handling various waste streams, such as textiles, home hazardous trash, and electronic garbage (Das et al. [55]; Ghinea et al. [56]). Public education campaigns, extended producer responsibility programs, and targeted collection activities are standard components of successful management plans. Research indicates that cultural, economic, and legal variables can substantially impact the content of miscellaneous garbage, as demonstrated by Chen et al. [57]. As a result, generalisations regarding the prevalence of various waste should be regarded with caution. Education and awareness are crucial in promoting behavioural change towards sustainable waste management practices. Community outreach programs, educational workshops, school curriculum integration, and multimedia campaigns can all help raise awareness about the environmental impacts of waste, empower individuals to make informed choices, and foster a culture of waste reduction, reuse, and recycling.

From the results presented in Table 4, a correlation coefficient of 0.531 indicates a partial positive correlation. This implies that residents' awareness of waste and its impacts environment impacts is partially correlated with the municipality's effectiveness of waste policies and laws. A significant value of 0.000 also indicates that the correlation is highly significant; thus, if policies are highly effective, they will create awareness of waste and its impact on the municipality. Raising awareness about the impact of improper solid waste practices is crucial for promoting good waste management practices. The findings imply that well-designed waste policies and laws are crucial in shaping residents' awareness and behaviour towards waste management. Effective policies, such as those promoting recycling, implementing waste segregation practices, and enforcing penalties for illegal dumping, can raise awareness and foster responsible waste disposal behaviours among the population. The partial positive correlation suggests a mutually reinforcing relationship between awareness and policy effectiveness. As residents become more aware of waste issues, they may pressure policymakers to implement and enforce robust waste management policies. Conversely, effective policies can enhance awareness by providing clear guidelines and incentives for sustainable waste practices. Effective waste management policies are essential to address the growing challenges of waste generation, environmental degradation, and resource depletion, as stated by Sukholthaman and Shirahada [58]. Policies should prioritise the waste hierarchy, which ranks waste management strategies from most to least preferred: prevention, reduction, reuse, recycling, recovery, and disposal. Policies should encourage practices that minimize waste generation and promote resource efficiency.

**Table 4:** Pearson correlation of waste policies and awareness of waste impacts.

		Non-effectiveness of waste policies and laws	Awareness of waste and its impact
Non-effectiveness of waste policies and laws	Pearson Correlation	1	0.531^a^
	Sig. (2-tailed)		0.000
	N	394	394
Awareness of waste and its impact	Pearson Correlation	0.531^a^	1
	Sig. (2-tailed)	0.000	
	N	394	394

a Correlation is significant at the 0.01 level (2-tailed).

The results in Table 5 indicate a correlation between respondents’ awareness of improper solid waste disposal and their willingness to adopt good solid waste practices.

**Table 5:** Awareness of improper disposal and willingness to adopt good waste management practices.

		Indiscriminate disposal of waste and climate change	Willingness to adopt good waste disposal practices
Indiscriminate disposal of waste and climate change	Pearson Correlation	1	0.594^a^
	Sig. (2-tailed)		0.000
	N	394	394
Willingness to adopt good waste disposal practices	Pearson Correlation	0.594^a^	1
	Sig. (2-tailed)	0.000	
	N	394	394

a Correlation is significant at the 0.01 level (2-tailed).

A coefficient of 0.594 indicates a moderately positive correlation between awareness of improper waste disposal and climate change and willingness to adopt good waste management strategies to mitigate climate change. Thus, residents’ awareness of how improper waste disposal contributes to global warming has a significance on their willingness to adopt good waste management practices to mitigate the release of GHGs (since the significance is less than 0.01).

Higher-income individuals may have more resources to invest in waste management infrastructure. Therefore, the researcher ascertained the relationship between households’ income and willingness to adopt good waste management practices. The outcome is presented in Table 6.

**Table 6:** Pearson correlation of willingness to adopt good waste strategies and income of respondents.

		Willingness	Income
Willingness	Pearson Correlation	1	−0.045
	Sig. (2-tailed)		0.370
	N	394	394
Income	Pearson Correlation	−0.045	1
	Sig. (2-tailed)	0.370	
	N	394	394

From the results presented in Tables 6, it can be deduced that there was a weak relationship between respondent's total income and their willingness to adopt good waste management practices (*r* = *-0.045).* This suggests that income level alone is not a strong predictor of an individual's willingness to engage in sustainable waste management behaviours. The p-values (0.370) for both correlations are more significant than the conventional significance level of 0.05. This indicates that the observed correlations are not statistically significant at the 95 % confidence level, meaning that any observed relationship between willingness and income could likely be due to chance. The non-significant correlation suggests that income level alone does not influence willingness to adopt good waste management practices. This implies that individuals from various income brackets may possess similar levels of willingness to engage in sustainable waste management behaviours. Municipalities and organisations promoting waste management initiatives should adopt inclusive strategies that target individuals across all income levels. Programs should focus on raising awareness, providing education, and offering practical solutions that appeal to diverse socioeconomic backgrounds. Policymakers should prioritise policies and programs that ensure equitable access to waste management services and infrastructure, regardless of income level. This may involve subsidising waste collection services, implementing recycling incentive programs, or providing resources for community-based waste reduction initiatives. According to Talisay et al. [59], environmentally friendly citizens and families with high incomes do not always conduct proper solid waste management. As a result, both the wealthy and the impoverished start disposing of waste improperly. To capitalise on this correlation (see Table 6), municipalities should prioritise community engagement and participation in waste management initiatives. By involving residents in decision-making processes, fostering dialogue between stakeholders, and soliciting feedback on policy implementation, municipalities can create a sense of ownership and accountability among residents, leading to greater compliance with waste management regulations.

From Table 6, policymakers should prioritise policies and programs that ensure equitable access to waste management services and infrastructure, regardless of income level. This may involve subsidising waste collection services, implementing recycling incentive programs, or providing resources for community-based waste reduction initiatives. Since income level alone does not determine willingness to participate in waste management efforts, community engagement becomes crucial. Encouraging community involvement through outreach events, volunteer opportunities, and participatory decision-making processes can foster a sense of ownership and collective responsibility for waste management. While income may not directly influence willingness to engage in waste management, other socioeconomic factors such as education, access to information, and cultural norms may play significant roles (Echegaray and Hansstein [60]).

### 3.2 Household waste disposal methods and their preferred methods of waste disposal

**Fig. 3:**
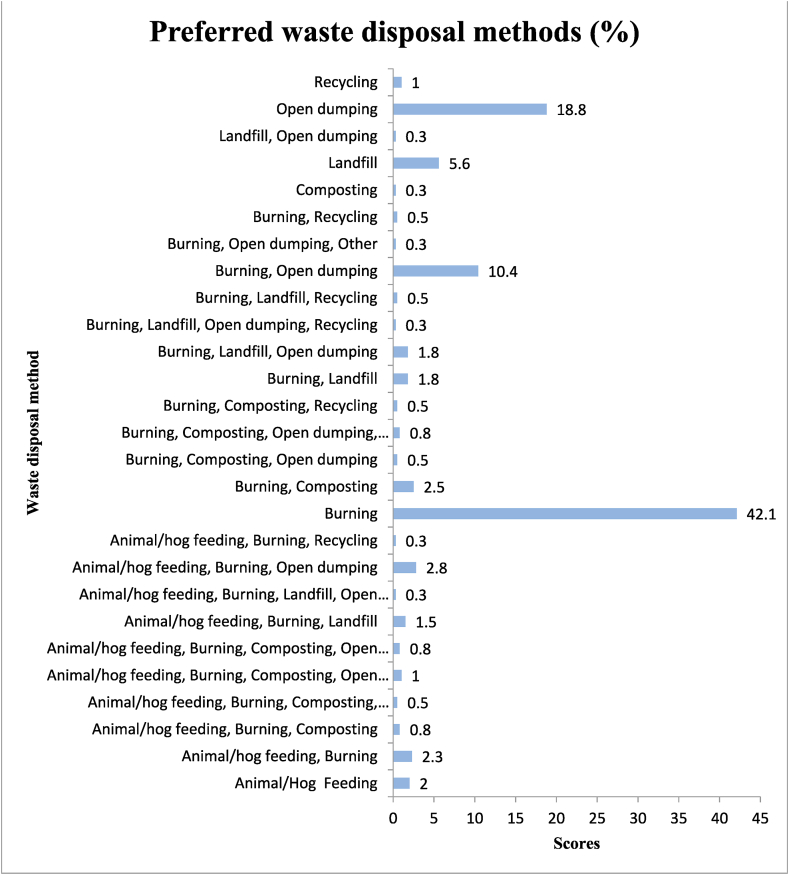
Preferred waste disposal methods.

**Fig. 4:**
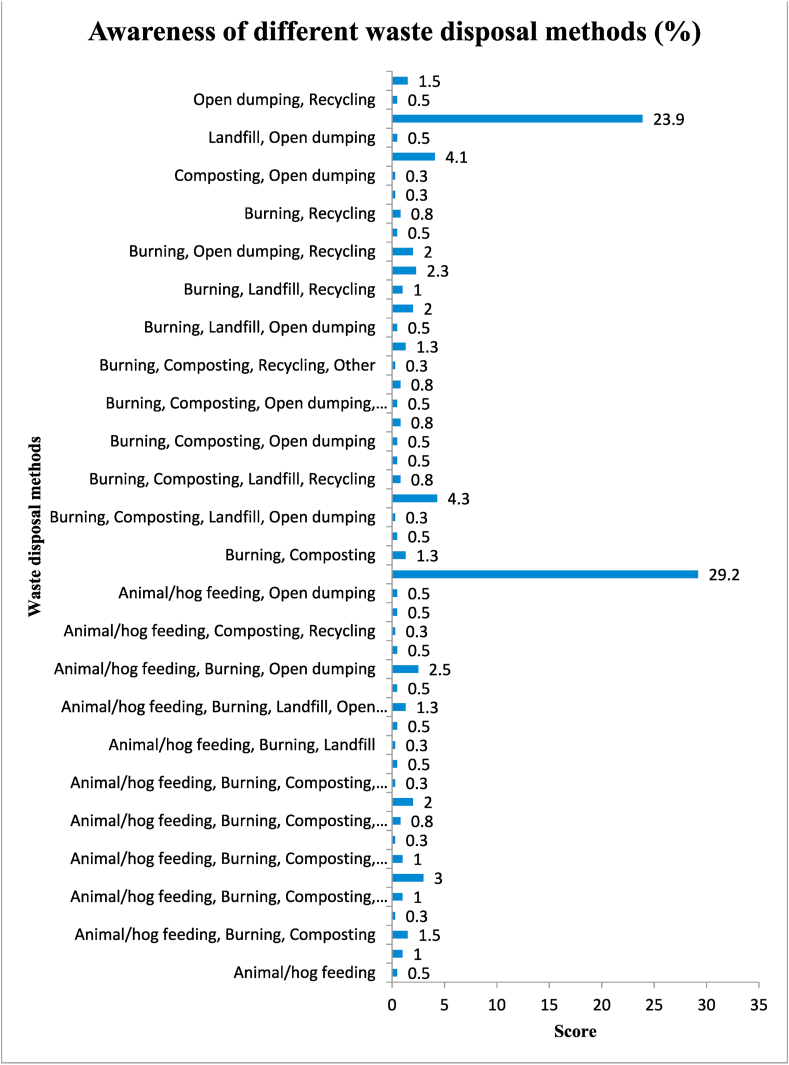
Awareness of different waste disposal methods.

Household waste disposal methods are essential for several reasons. Proper waste disposal contributes to a clean and aesthetically pleasing environment. Litter and improperly discarded waste can negatively impact the appearance of communities and recreational areas (Gwada et al. [61]). By following proper disposal methods, individuals contribute to broader environmental goals, such as reducing carbon emissions, conserving natural resources, and promoting a more sustainable way of living (Satterthwaite [62]). Hence, the researcher ascertained the residents’ disposal methods and their awareness of various traditional waste methods and the results are presented in Fig. 3, Fig. 4. Solid waste is disposed of in several ways, but the primary method adopted and preferred by households and other generators is open burning and dumping. The results presented in Fig. 3 provide details of refuse disposal (preferred) methods in the Kassenan Nankana Municipality.

The bars presented in Fig. 3 above indicate that most households' waste generators patronise open burning and dumping, which account for 42.1 % and 18.8 %, respectively. These phenomena could be explained by the urbanised nature of the Municipality and the absence or lack of public dumps and refuse containers for residential areas, which is in line with a study done by (Addaney and Oppong [63]) in the Kasoa Township. This also can result from difficulties obtaining dust bins from the municipality (Table 7). During the focus group discussion with 36 participants, it was observed that acquiring a dustbin is difficult and expensive. Some reasons residents patronise burning are its affordability, prevention of the spread of diseases, improvement of aesthetic environs, sporadic/isolated settlement and weak law enforcement. Detailed descriptions of residents’ reasons are presented in Table 7 below. Although residents were aware of the negative impacts of burning on the environment, they still preferred it to other forms of waste management. This finding is in line with the study by Essaw and Sasu [30], who made a case that even while people may be aware of the harmful impacts of burning, they will still do it because they believe it is a better option than other methods of managing waste, such as burying it in the ground.

**Table 7:** Focus group discussion on why residents patronise open burning and dumping.

Theme	Repeated critical/implicit statements	Key Excerpts
Very Affordable	• Burning is cheap • *Does not demand payment* • Anytime and any place • Easy to practice	*“I can burn my waste anytime I want, and it is so easy*” *(Respondents II, III, IV)* *“I do not have to think about any form of payment” (Respondent III)* *“Acquiring a dust bin is expensive; even just common registration is 50 cedis.”*
Way of preventing the spread of diseases	• Burning is momentary • Unpleasant odour	*“When I burn, no flies will have the chance to step on piled waste" (Respondent V).* “Burning reduces the probability of degradable waste decomposing to emit odour” (Respondent II)
Improve aesthetic value	• Little carried by the wind	*“When I leave my waste in an uncovered bin, they are easily carried by the wind, dirtying the environment” (Respondent II)*
Sporadic/isolated settlement	• Scattered settlement encourages open dumping.	*“The nature of houses in many rural settlements are scattered. This reduces monitoring by neighbours*, *encouraging open dumping” (Respondent I).*
Weak enforcement of laws	• Weak law enforcement	*“You will report someone to the Municipal Sanitation officer to be taken on, but to no avail. They will tell you they had a command from above to let the person go.” (Respondent I)* *“The penalty unit for sanitation offences is too small. I can waste fuel up and down to prosecute someone for something small” (Respondent II)*

Though residents may prefer open burning and dumping of waste for several reasons, it is essential to note that burning waste, especially in an uncontrolled or inefficient manner, can have negative environmental and health impacts (Abubakar et al. [64]; Mor and Ravindra [65]). Open dumps release methane from the decomposition of biodegradable waste under anaerobic conditions. Methane causes fires and explosions and significantly contributes to global warming (Aziz et al. [66]). For open dumping, discarded tyres at dumps collect water, allowing mosquitoes to breed, increasing the risk of diseases such as malaria, dengue and West Nile fever (Krystosik et al. [67]; Agyemang-Badu et al. [68]). Uncontrolled waste burning at dump sites releases fine particles, which significantly cause respiratory disease and smog (Sridevi et al. [69]). There are also problems associated with odour and migration of leachates to receiving waters from open dumping (Srivastava et al. [70]). According to Hajam et al. [71]), burning generates environmentally unfriendly molecules that have a negative impact on the biosphere and the atmosphere.

The findings (Fig. 3, Fig. 4) with their reasons (Table 7) for other forms of waste disposal methods are in line with studies conducted by Nyumah et al. [72] and Garcia et al. [38]. Awafo et al. [73] asserted that residents in peri-urban areas preferred open burning due to limited waste collection services and a lack of convenient disposal options. Again, Alhassan et al. [74] opined that residents' preference for waste dumping in open spaces is due to traditional beliefs regarding waste disposal. The lack of waste collection infrastructure led residents to resort to waste dumping, favouring convenience over sustainable waste management practices (Quaye et al. [75]). Limited access to waste collection services resulted in residents resorting to open burning as a quick disposal method (Mensah et al. [76]). Residents' preference for waste dumping was attributed to irregular waste collection services and inadequate disposal infrastructure (Obeng et al. [77]). All these studies reveal residents' preferences for waste dumping and open burning in specific contexts, highlighting the reasons behind such preferences and the challenges related to waste management infrastructure and services, which are also in line with the findings of this study.

The bars presented in Fig. 4 demonstrate residents’ awareness of different forms of waste practices. The results show that residents are at least aware of the various types of waste disposal strategies. The most dominant ones were burning and open dumping, with percentages of 29.2 and 23.9, respectively.

#### 3.2.1 Traditional waste management in Kassena Nankana

There is no waste segregation system either at the household or disposal points. Both household and municipal waste are collected together and sent to either skip for disposal or dump. Many households dump their domestic waste in pits or use it for composting in backyard farms. The Communal Collection System (CCS) entails placing metal containers at predetermined locations to serve several homes in that neighbourhood. After the waste is disposed of at the final sites, it is either burned or compacted. On a separate contract, two private WM companies, the Environmental Service Provider Association (ESPA) and Zoomlion Ghana Limited (ZLG), provide WM services to the NM.

#### 3.2.2 Description of existing sanitation and location of skips

**Fig. 5:**
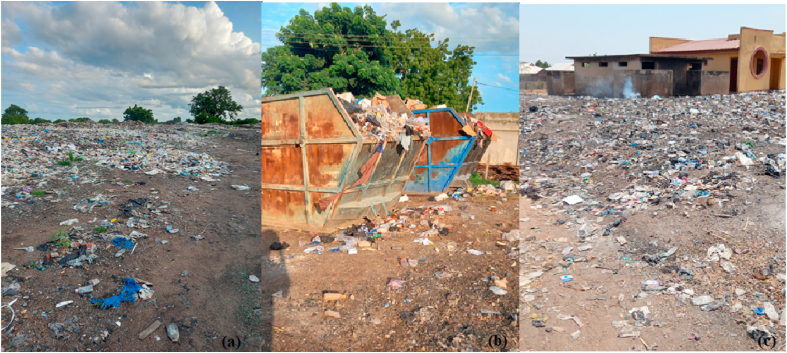
Final disposal site and location of skips.

**Fig. 6:**
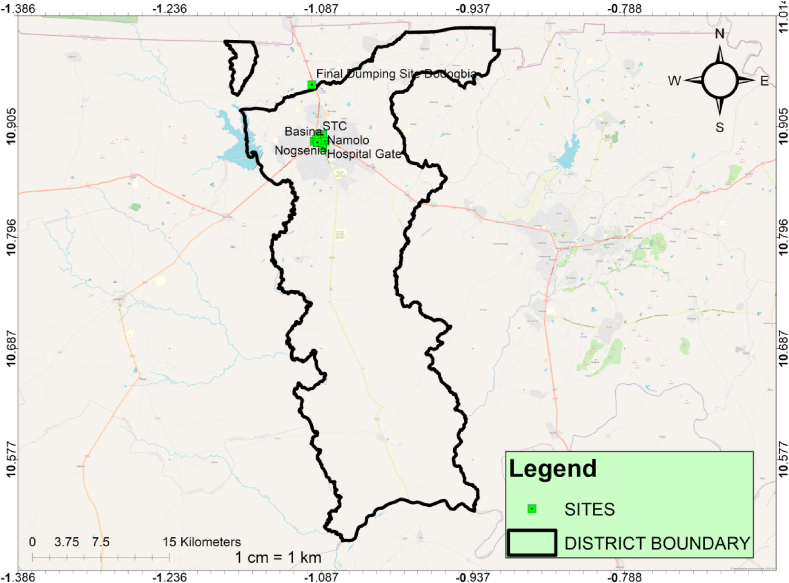
Location of skips [Map designed with ArcMap 10.7].

Environmental cleanliness is critical in promoting people's welfare, worker efficiency, and health. Nonetheless, there is still much room for improvement in terms of hygiene. Most of the Municipality's insufficient sanitation services are found in urban areas, with the remainder in marketplaces and educational institutions. The municipality has one (1) final disposal site (Fig. 5a) outside the municipality (Located in the Kassena Nankana West District), eight (8) 10 m^3^ refuse containers (skip containers; Fig. 5b and c), and seven hundred and forty –eight (748) 240L refuse containers (Kassena Nankana Municipal Assembly [78]). A dump is a location where solid waste is dumped with no following rules governing the environment. A landfill is "a structure used for the disposal of solid waste from commercial, governmental, and other sources; sanitary landfills are those managed to safeguard the environment standards." (Nanda and Berruti [79]). Generally speaking, sanitary sites are public areas used as landfills in towns. There are instances when they are given communal skips, which are enormous steel containers used for collecting and transporting rubbish regularly, and when they are just open spaces for disposing of waste. (Ampofo et al. [26]). According to Ampofo et al. [80], these are often the locations of public restrooms in neighbourhoods without indoor plumbing. These factors, which show the rate of garbage disposal, the amount of waste disposed of by households, and the collection rates by municipal authorities, help evaluate the effectiveness of waste management in the municipality. It is also helpful in determining how effective the current management system is in terms of the number of sites accessible, the population of a community, and the amount of garbage produced. Geographic coordinates of the location (Fig. 6) of eight (8) skip containers and one (1) open dump were taken from 8 (eight) suburbs within the Navrongo Central Zone since all the skips are in that zonal council. Before Paga, a final waste disposal site outside of Navrongo lacks waste treatment facilities, where the municipality disposes of its final waste.

The absence of a waste segregation system at both household and disposal points has significant implications for the study's findings. Firstly, it indicates a lack of organised waste management practices in the municipality, which aligns with the findings highlighting challenges in waste management. This lack of segregation can lead to waste handling and disposal inefficiencies, contributing to environmental pollution and health hazards (Nandan et al. [81]). Overall, the absence of waste segregation systems necessitates comprehensive interventions and policy reforms to promote sustainable waste management practices in the municipality. This includes implementing waste segregation policies, strengthening regulatory frameworks, and fostering community engagement to support collective action towards improved waste management.

The inadequate sanitation services and sites (Fig. 5, Fig. 6) highlight a pressing need for improved waste management practices to ensure environmental cleanliness and public health. This underscores the relevance and urgency of the study's focus on understanding the municipality's perceptions of waste management behaviour. The presence of only one final disposal site outside the municipality, coupled with the limited number of skip and refuse containers, indicates infrastructural challenges in waste disposal. According to Ampofo et al. [26], these challenges can impact the effectiveness of waste management systems and contribute to environmental pollution. The existence of open dumps without environmental regulations poses health risks and emphasises the need for regulatory measures to ensure sanitary waste disposal. Additionally, the geographic distribution of skip containers and open dumps within the Navrongo Central Zone highlights spatial disparities in waste management infrastructure. This spatial analysis can inform policymakers and stakeholders about areas with inadequate waste management facilities, guiding targeted interventions to improve waste management practices.

### 3.3 Household satisfaction on waste management by the assembly and companies

By describing the overall flow situation regarding solid waste management in Kassena Nankana Municipality in terms of the population's satisfaction with the quality of collection services at their zonal councils, this study aimed to provide enough evidence for decision-makers to interpret comprehensive understanding and utilise it to their communities through the implementation of relevant policies to control Municipal Solid Waste activities. Local sustainability of waste management procedures will be made possible by an appreciation of local conditions supported by the satisfaction of the populace (Al-Khatib, [82]). The results of the households' satisfaction with waste in the municipality are presented in Table 8 below.

**Table 8:** Household satisfaction on waste management by the assembly and companies.

Statements		SA (5)	A (4)	NS (3)	D (2)	SD (1)	SI
At this point, waste management in the municipality needs to be changed drastically.	FR	147	113	40	55	39	67.4
	PR	37.3	28.7	10.2	14.0	9.9	
There must be a revised waste management programs that will provide residents with better options for managing waste.	FR	141	129	43	52	29	69.1
	PR	35.8	32.7	10.9	13.2	7.4	
I am aware of waste management policies and laws in the municipality.	FR	30	110	81	98	75	45.1
	PR	7.6	27.9	20.6	24.9	19.0	
Waste management laws and policies in the municipality are not effective.	FR	137	90	113	37	17	68.6
	PR	34.8	22.8	28.7	9.4	4.3	
Waste management companies better meet residents' demands on waste management in the municipality.	FR	10	53	109	137	85	35.2
	PR	2.5	13.5	27.7	34.8	21.6	

FR; Frequency Responses, PR: Percentage Responses, SI; Severity Index.

**Table 9:** Residents' knowledge about the impact of waste.

Statements		SA (5)	A (4)	NS (3)	D (2)	SD (1)	SI
I am aware of both the direct and indirect effects of waste on the environment.	FR	133	142	54	48	17	70.7
	PR	33.8	36.0	13.7	12.2	4.3	
I am aware that improper waste disposal can lead to land and water pollution.	FR	194	144	22	24	10	81.0
	PR	49.2	36.5	5.6	6.1	2.5	
Indiscriminate disposal of waste can contribute to climate change and global warming.	FR	140	142	57	25	30	71.4
	PR	35.5	36.0	14.5	6.3	7.6	
Improper disposal of waste can cause severe contagious or infectious diseases.	FR	195	161	6	14	18	81.8
	PR	49.5	40.9	1.5	3.6	4.6	
Loss of biodiversity is partly a result of improper waste management.	FR	72	183	84	47	8	66.8
	PR	18.3	46.4	21.3	11.9	2.0	
I am willing to adapt and adopt a favourable waste management system that will not adversely affect the environment and mitigate climate change.	FR	157	137	78	12	10	76.6
	PR	39.8	34.8	19.8	3.0	2.5	

FR; Frequency Responses, PR: Percentage Responses, SI; Severity Index.

From the results presented in Table 8 above, it can be seen that most respondents with a severity index (SI) of 67.4 agree that waste management in the municipality needs to be changed drastically. With a SI of 35.2, many respondents disagree that the companies and the municipality do not better meet their demands concerning waste management. Residents might not be happy with municipal waste management strategies for various reasons. Upon further probing, the study found inadequate collection receptacles and difficulty in assessing receptacle bins. This study aligns with Ezebilo [83], who asserted that if patronising municipal waste management is too high, most residents will not patronise.

Furthermore, residents responded that the policies and laws guiding waste management in the municipality are ineffective, with a SI of 68.6. This might be why many of the respondents are neutral (SI = 45.1) about these policies in the municipality. Thus, residents lack much information on waste policies and laws. Additionally, citizens' opinions of the law, which depend on their awareness of the laws and regulations, will determine how likely they are to follow them (Cavaliere et al. [52]). The expert interview revealed several sanctioning mechanisms, such as fines associated with unlawful garbage disposal, are in place for norms and regulations; according to a study by Babayemi et al. [84], respondents I and II (Table 10) did note that some of the laws and regulations would be challenging to implement since local authorities cannot track and enforce the legislation. Effective waste policies ensure proper disposal and treatment of waste, minimising pollution and its impact on local ecosystems, air and water. These findings align with Lissah et al. [85], who indicated in their research that residents expressed dissatisfaction with waste management services, citing irregular collection, inadequate bins, and insufficient education programs by waste management companies.

**Table 10:** Assembly and companies satisfaction and management of waste.

Theme	Repeated critical/implicit statements	Key Excerpts
Improvement of waste management in the municipality	There has been improvement in the municipality (Respondent I) *Residents are yielding to private waste management companies (*Respondents I and II)	*“Since I assumed office, waste management in the municipality has strategically improved*” *(Respondents I and II.)* *“Many of the residents are now patronising and purchasing waste bins from Zoomlion” (Respondents I and II)*
Satisfaction with the waste management system in the municipality	Residents are doing their part (Respondents I) Improved waste management (Respondents I and II)	*“If I am to score the satisfaction on a scale of 10, I will give a satisfaction rate of 8" (Respondent I).* “*I will score my satisfaction level 5 out of 10” (Respondent II).*
Enforcement of sanitation laws in the municipality	Poor enforcement of law (Respondents I, II and III)	*“You will report someone to the Municipal Sanitation officer to be taken on, but to no avail. They will tell you they had a command from above to let the person go.” (Respondent I)* *“The penalty unit for sanitation offences is too small. I can waste fuel up and down to prosecute someone for something small” (Respondent II)*
Intervention and policies to improve waste management	Enforcement of laws, Education, and patronisation of residents (Respondents I and IV) Showing respect, enforcement of the law, and provision of waste bins (Respondents I, II, III and VI)	*"To prevent us from living with our waste in our houses, the district assembly must supply the area with waste bins that can be evacuated whenever they become filled." (Respondent I).* “*The penalty unit for sanitation offences must be increased*” (I). “*Education is also key in improving waste management in the municipality.”*

### 3.4 Residents’ awareness about the impacts of improper disposal of waste

Behavioural or psychological treatments typically entail giving actors in a scenario awareness to alter their behaviour. There are numerous ways to obtain this kind of data. For instance, knowledge or awareness of the issue enables communities to characterise pollution in their own words, as does knowledge of its solutions and effects (Heidbreder [86]). Hence, the researcher ascertained the residents’ knowledge and awareness of the impacts of improper waste disposal on their environment.

With a SI of 70.7 and 81.0, most respondents are aware of both the direct and indirect effects of waste on the environment and that improper disposal of waste can lead to pollution respectively. Again, respondents agree that improper waste disposal contributes to climate change and infectious diseases. Implementing appropriate and sustainable waste management systems requires changing people's attitudes toward garbage and raising public consciousness (Kumar et al. [87]). Thus, awareness and willingness (SI = 76.6) go together (r = 0.594, Table 5); so long as residents are aware of the impact of waste, they are willing to adopt waste strategies that can mitigate or reduce climate change. The mindsets and actions of individuals regarding the management of waste may, therefore, be influenced by their comprehension of the effects of waste and the appropriate waste management procedures. Thus, to address the waste management dilemma, individual or group understanding and attitudes regarding trash generation and management are crucial (Essaw and Sasu [30]). Most respondents (Table 9) are aware of both the direct and indirect effects of waste on the environment and that improper disposal of waste can lead to pollution. A study by Chen and Chang [88] found that while respondents exhibited a high level of knowledge about waste management's environmental impacts, their actual waste disposal practices did not consistently align with this knowledge. This discrepancy between knowledge and behaviour was attributed to various factors, including convenience, lack of proper infrastructure, and customary practices. Similarly, the research conducted by Al-Khatib et al. [89] revealed a notable gap between residents' attitudes and their waste disposal behaviours. Despite having a positive attitude towards waste management practices and understanding the potential environmental consequences, many respondents continued to dispose of waste improperly due to cultural norms, lack of enforcement of regulations, and inadequate waste management infrastructure. These studies demonstrate that knowledge alone does not necessarily translate into proper waste disposal practices. Cultural, infrastructural, and social factors significantly influence residents' behaviours, contributing to the persistent practice of improper waste disposal despite their awareness of its impacts.

**Fig. 7:**
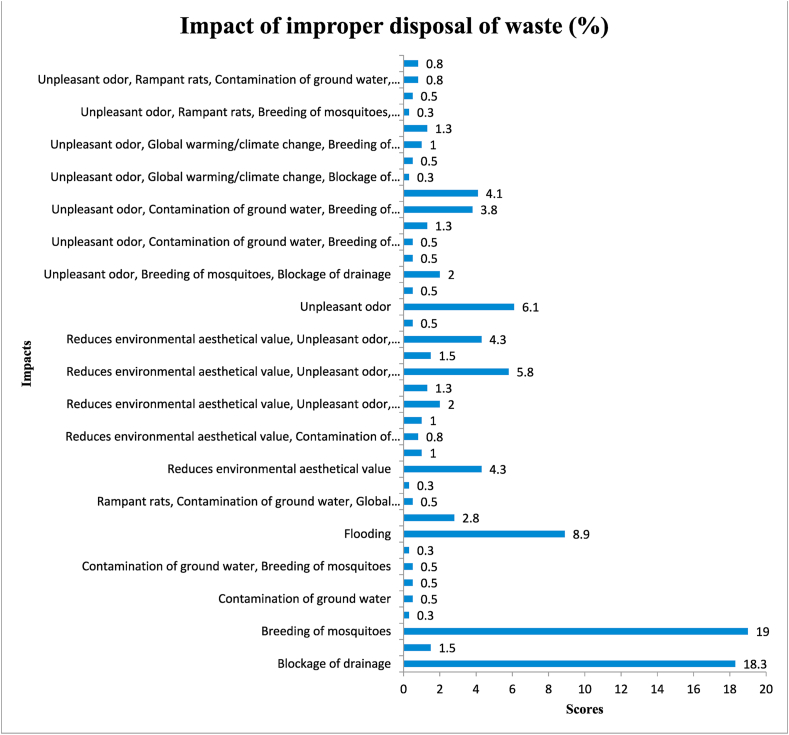
Impact of improper disposal of waste.

Fig. 7 demonstrates the different environmental risks that residents have identified posed by improper disposal of solid waste. Some of the grievous dangers posed by improper waste disposal included breeding grounds for mosquitoes (19 %), blocking drainage systems (18.3 %), flooding (8.9 %) and others. These results are consistent with the findings of Cudjoe [90], which indicated that waste choked a city's drainage systems, preventing torrential rains from leaving the city and thereby causing flooding consistently. Additionally, Surendran et al. [91] provided additional evidence supporting the study's conclusions by showing that blocked drainage systems also result in pools of stagnant water, which facilitate the breeding of mosquitoes and other animals (insects) in urban areas and may be a source of disease transmission. Therefore, sufficient focus should be given to waste disposal to prevent these harmful effects of waste on the environment.

**Fig. 8:**
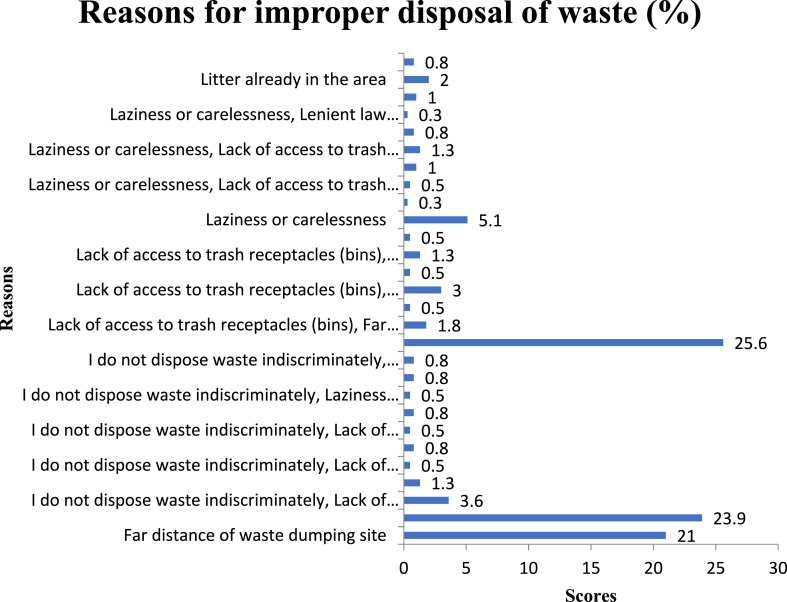
Reasons for improper disposal of waste.

The researcher ascertained the reasons behind the improper disposal of waste. The bars in Fig. 8 demonstrate various reasons behind some residents’ improper waste disposal. Among the dominant reasons are lack of access to trash receptacles (25.6 %) and a far distance from waste dumping sites (23.9 %). These findings are in line with Puopiel [31], who also asserted lack of dustbins or skips, lack of dumpsites, distance of dumping sites, and higher charges from waste management firms providing door-to-door service are some of the challenges in managing municipal waste in the Tamale Metropolitan Area. When these difficulties are persistently ignored, households resort to improper disposal techniques, including filling gutters, roadsides, spaces behind homes, water bodies, and any available open areas. This could be the cause of the rise in polyethene bags and pure water sachets in the Municipality, as well as the fact that 81.5 % of respondents (Table 2) thought there was an issue with handling waste there.

### 3.5 The Assembly's satisfactions on waste management by the citizens/households

Informant/expert interviews (with 4 persons) and focus group discussions were conducted with respondents to gather information and ascertain the perceptions of unit heads at the district levels who are directly or indirectly influencing elements of waste management in the municipality. This helps with the triangulation and improves the validity of the study's overall results (Natow [92]). The interview respondents include opinion leaders from the Municipality (respondents in the Zoomlion and the Municipal Environment and Health Unit offices). Considerations regarding respondents' anonymity led to the withholding of their names. Nonetheless, each participant is assigned a distinctive Roman numeral ID to make documentation and interpretation of the findings easier.

The study also found that the effectiveness of waste control regulations being applied at the level of rural communities is influenced by the cooperation and efficiency of regulatory organisations, such as the Environmental Protection Agency and the Environmental Health Department of the Kassena Nankana Municipality (Fobil et al. [93]). A comparable study has demonstrated that WM in societies is adversely impacted by the regulatory authorities' incompetence and lack of cooperation (Lissah et al. [85]). Enhancements in collaboration and capacity building among these organisations are necessary to address the inadequate WM procedures throughout the research region and to improve the effectiveness of WM and environmental hygiene.

Even though most participants claimed to be fully aware of WM rules at the municipal and community levels, there was no much effort to implementating these laws. Whenever it comes to WM, the municipal assembly is only particularly picky about the city's densely populated areas or what might be considered larger cities. The shortage of environmental awareness-building and education about the potentially severe consequences of improper trash disposal may contribute to negative behaviours and emotions in rural communities. The issue of people's negative attitudes and low-risk perceptions was not exceptional in the current study since prior research indicates that local government officials in most developing towns have similar challenges in controlling garbage (Lissah et al. [85]). Table 10 presents themes and key excerpts related to assembly and company satisfaction with waste management, focusing on improving waste management, satisfaction with the waste management system, enforcement of sanitation laws, and interventions and policies to improve waste management.

Respondents reported progress in municipal efforts to address waste challenges. The willingness of residents to engage with private waste management companies like Zoomlion suggests a growing acceptance of external service providers, potentially relieving some burden from the municipal authorities. This implies that the Municipality should continue investing in waste management infrastructure and partnerships with private companies to sustain and build upon the observed improvements in line with (Bhuiyan and Islam [94]. Concerning satisfaction with the Waste Management System, there are mixed levels of satisfaction among respondents, indicating varying perceptions of the effectiveness of the waste management system. While some respondents express high satisfaction levels, others indicate lower satisfaction levels, highlighting areas for improvement. This suggests that the municipality should regularly assess citizen satisfaction and address identified gaps to enhance overall satisfaction levels and public confidence in waste management services (Bhuiyan and Islam [94]).

In response to companies’ satisfaction with the enforcement of sanitation laws in the Municipality, there are reports of poor enforcement of sanitation laws. These suggest challenges in regulatory compliance and law enforcement within the municipality. Instances of leniency and inadequate penalties for sanitation offences counteract the effectiveness of enforcement efforts. This implies that Municipal authorities need to strengthen enforcement mechanisms, increase penalties for sanitation offences, and ensure consistent application of regulations to deter non-compliance and improve overall sanitation standards.

Respondents highlight the importance of varied interventions, including enforcement of laws, education initiatives, provision of waste bins, and increasing penalties for offences. The suggested calls for respect for regulations, education campaigns, and improved waste infrastructure give extra weight to the need for comprehensive approaches to address waste management challenges. This implies that Municipalities should adopt integrated strategies that combine regulatory measures with public education, infrastructure development, and community engagement to foster sustainable waste management practices and behaviour change (Zorpas [95]). In summary, the implications of Table 10 emphasise the importance of ongoing efforts to improve waste management systems, address enforcement challenges, and implement various interventions to enhance the overall satisfaction and effectiveness of waste management services and authorities within the municipality (Raghu and Rodrigues [15]).

### 3.6 Contribution to knowledge

**Fig. 9:**
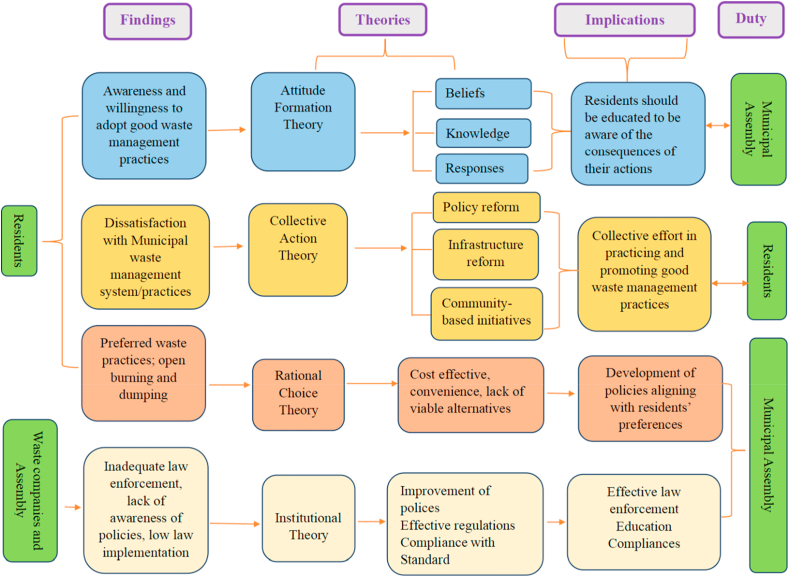
Summary of Study's findings to contributions of theories.

The study advances our current understanding of the subject by offering insights into the waste management methods, challenges and behaviours in the Kassena Nankana East Municipality. Using an extensive examination of socio-demographic variables, techniques for disposing of waste, degrees of awareness and preferences, and contentment with waste management services, the research illuminates the complex relationships that impact waste management practices and consequences. Using theoretical frameworks including rational choice theory, attitude formation theory, collective action theory, and institutional theory, the research provides a more profound comprehension of the variables influencing individual and group decision-making processes concerning waste disposal (see Fig. 9). The results emphasise how crucial it is to solve institutional shortcomings, encourage community involvement, and provide focused interventions to advance sustainable waste management techniques.

The findings regarding residents' awareness of waste management issues and their willingness to adopt good waste management practices align with attitude formation theory. According to this theory, attitudes are formed through cognitive processes involving beliefs, knowledge, and affective responses towards a particular object or behaviour, as portrayed by Cacioppo et al. [96]. The study's results suggest that residents' awareness of the environmental impacts of improper waste disposal influences their attitudes towards waste management practices. As residents become more aware of the consequences of their actions, they are more likely to develop positive attitudes towards adopting sustainable waste management behaviours.

Collective action theory posits that individuals collectively work towards achieving common goals or addressing shared concerns, as proposed by Olson (Ntamu et al. [97]). The findings related to residents' dissatisfaction with waste management practices, calls for improved enforcement of sanitation laws, and support for many-sided interventions align with collective action theory. Residents' perceptions of inadequate waste management services and enforcement mechanisms motivate collective action to advocate for policy reforms, infrastructure improvements, and community-based initiatives to address waste management challenges. The study highlights the importance of collective efforts in promoting sustainable waste management practices and fostering community engagement. From a theoretical perspective, residents’ lack of waste segregation reflects the limitations of collective action theory, which emphasises the importance of coordinated efforts by individuals or groups to address common issues like waste management. The absence of a segregation system suggests a lack of collective action and coordination among stakeholders, hindering effective waste management practices.

Rational choice theory suggests that individuals make decisions based on a rational assessment of costs and benefits, aiming to maximise their utility, as Becker postulates (Byron [98]). In this study's waste management context, the findings related to residents' preferences for open burning and dumping despite awareness of the negative consequences can be interpreted through rational choice theory. Residents may opt for these disposal methods due to perceived cost-effectiveness, convenience, or lack of viable alternatives, even though they know the environmental and health risks associated with such practices. Understanding residents' decision-making processes within the framework of rational choice theory can inform the design of interventions and policies that align with their preferences while promoting sustainable waste management behaviours. Furthermore, the reliance on private waste management companies for waste collection services suggests a reliance on market-driven solutions, reflecting aspects of rational choice theory. However, without proper regulations and enforcement mechanisms, such as waste segregation requirements, these private companies may prioritise profit over environmental concerns, leading to suboptimal waste management outcomes.

Institutional theories emphasise the role of formal and informal institutions in shaping individuals' behaviors and organisational practices according to (Peters [99]). The study's findings regarding residents' perceptions of ineffective waste management policies and enforcement mechanisms align with institutional theory. Inadequate enforcement of sanitation laws, lack of awareness about existing policies, and challenges in implementation reflect institutional deficiencies that hinder effective waste management. Addressing these institutional barriers requires reforms in governance structures, policy frameworks, and institutional capacities to ensure more effective regulation, enforcement, and compliance with waste management standards.

The contributions of the findings to these theories support the complexity of waste management dynamics and the need for various approaches that consider individual attitudes, collective action, rational decision-making processes, and institutional contexts (Raghu and Rodrigues [15]). Understanding how these theories intersect with waste management practices can inform the development of more effective strategies and policies to promote sustainable waste management behaviours. By aligning interventions with theoretical frameworks such as attitude formation theory, collective action theory, rational choice theory, and institutional theory, policymakers and practitioners can design targeted interventions that address fundamental drivers of behaviour change, mobilise collective action, incentivise rational decision-making, and strengthen institutional capacities for effective waste management governance (Popa [100]). Ultimately, integrating theoretical insights into practical interventions can lead to more sustainable waste management practices, enhance environmental stewardship, and improve the well-being of communities by mitigating the adverse impacts of improper waste disposal.

## 4 Conclusion and recommendation

### 4.1 Conclusion

In conclusion, this study provides comprehensive insights into waste management practices within the Kassena Nankana East Municipality. The research highlights the significant influence of socio-demographic factors, such as education level and age, on waste disposal behaviours. A fundamental improvement over existing literature is the subtlety understanding of how demographic factors correlate with sustainable waste management practices, reinforcing findings by Ajzen [101] on attitude-behaviour consistency. The study's findings align with and expand upon previous research, such as Al-Khatib et al. [82,89], which highlighted discrepancies between residents' attitudes and their actual waste disposal behaviours due to cultural norms and infrastructure limitations. Incorporating local perceptions fosters community engagement and ownership of waste management initiatives. By respecting and integrating community beliefs and practices, sustainable solutions can be co-created with the residents. Through encouraging waste reduction practices, supporting composting initiatives, implementing recycling programs, ensuring equitable access to waste collection and disposal sites, and launching public awareness campaigns, significant contributions can be made to environmental theories/frameworks such as circular economy, ecological sustainability, waste hierarchy, environmental justice, and behavioural change. The study further elaborates on these discrepancies by incorporating theories of attitude formation and collective action, thus offering a more holistic view of the factors driving waste management behaviours. The study's novelty lies in its comprehensive analysis of waste management dynamics, its integration of theoretical frameworks, and its implications for policy and practice. By advancing our understanding of waste management behaviours and offering practical insights for addressing waste management challenges, the study significantly contributes to academic knowledge and real-world efforts to promote environmental sustainability.

Despite its contributions, the study acknowledges certain limitations. The cross-sectional nature of the survey restricts the ability to capture changes in attitudes and behaviours over time. Additionally, the reliance on self-reported data may introduce biases related to social desirability and recall accuracy. Future studies should consider longitudinal approaches and incorporate more diverse stakeholder voices to gain deeper insights into waste management's temporal dynamics and context-specific challenges. Future research should explore the effectiveness of targeted interventions based on the study's conceptual framework. Longitudinal studies could provide valuable data on the evolving perceptions and practices related to waste management. Additionally, integrating qualitative analyses will help capture the refinement perspectives of different community members, thus enhancing the relevance and applicability of the findings to policy and practice. Further investigation into the role of institutional frameworks and governance structures in shaping waste management outcomes is also recommended. This future research can build on the foundation laid by this study, contributing to more effective and sustainable waste management strategies that align with both local and global environmental goals.

### 4.2 Recommendation

Effective waste management is essential for promoting public health, preserving environmental quality, and advancing sustainable development goals. Building upon the study's findings in the Kassena Nankana East Municipality, several recommendations emerge to address the identified challenges and enhance waste management practices. These recommendations aim to improve waste collection infrastructure, promote sustainable waste disposal methods, strengthen enforcement of sanitation laws, invest in education and awareness programs, and facilitate stakeholder collaboration. By implementing these recommendations, stakeholders can work collaboratively to achieve more sustainable waste management outcomes, contributing to the overall well-being and prosperity of the municipality and aligning with the global agenda outlined in the Sustainable Development Goals.

1. Municipal authorities, Environmental Protection Agency (EPA), and Private Waste Management Companies should increase the number of trash receptacles in residential areas (both in rural and urban areas) to improve accessibility for residents. Furthermore, they should expand waste collection services to underserved communities and rural areas. This will ensure the contribution to SDG 11 (Sustainable Cities and Communities) to promote access to adequate, safe, and affordable housing and essential services for all.
2. Municipal Authorities, Environmental NGOs, and Community-Based Organisations (CBOs) must endeavour to implement public awareness campaigns promoting the use of sustainable waste disposal methods such as recycling, composting, and proper waste segregation. They can ensure the provision of incentives for households and businesses to adopt sustainable waste management practices. This ensures the contribution to SDG 12 (Responsible Consumption and Production) for achieving sustainable management and efficient use of natural resources.
3. Municipal Authorities, Law Enforcement Agencies and the Judicial System must increase enforcement efforts to deter illegal dumping and open waste burning through regular patrols and inspections. They should impose stricter penalties for sanitation offences to ensure compliance with waste management regulations. This will contribute to SDG 16 (Peace, Justice, and Strong Institutions). This will promote peaceful and inclusive societies for sustainable development, provide access to justice for all, and build effective, accountable, and inclusive institutions at all levels.
4. Municipal Authorities, Schools, Community Leaders and Media outlets should develop and implement educational programs in schools and communities to raise awareness about the environmental and health impacts of improper waste disposal. They should collaborate with local media outlets to disseminate information and resources on waste management practices. This contributes to SDG 4 (Quality Education), which ensures inclusive and equitable quality education and promotes lifelong learning opportunities for all.
5. The Waste Management Industry and the Municipality must invest in Waste-to-Energy facilities to convert non-recyclable waste into energy, reducing landfill reliance and providing a source of power. They should also upgrade and manage landfills to minimize environmental impacts, including employing technologies like landfill gas capture to reduce emissions. This will contribute to SDG 13: climate action to reduce climate change.

## Ethical consideration

Consent was obtained from all participants before their involvement in this study, ensuring compliance with ethical standards and safeguarding participants' rights and confidentiality. Additionally, all participants were informed about the objectives and or purposes of the study and asked for their voluntary participation.

## Data availability

Data will be made available by the authors upon request.

## CRediT authorship contribution statement

**Kwame Anokye:** Writing – review & editing, Writing – original draft, Validation, Methodology, Formal analysis, Conceptualization. **Sumaila Asaah Mohammed:** Writing – review & editing, Visualization, Supervision, Data curation, Conceptualization. **Portia Agyemang:** Writing – review & editing, Writing – original draft, Formal analysis, Data curation, Conceptualization. **Ahunoabobirim Bosompem Agya:** Writing – review & editing, Writing – original draft, Visualization, Methodology, Data curation, Conceptualization. **Ebenezer Ebo Yahans Amuah:** Writing – review & editing, Writing – original draft, Methodology. **Stephen Sodoke:** Writing – original draft, Formal analysis, Conceptualization. **Edmund Kude Diderutua:** Writing – original draft, Formal analysis, Data curation.

## Declaration of competing interest

The authors declare no known competing interests, receipt of funding, or personal relationships that could have influenced this research.
